# Activated platelets rescue apoptotic cells via paracrine activation of EGFR and DNA-dependent protein kinase

**DOI:** 10.1038/cddis.2014.373

**Published:** 2014-09-11

**Authors:** A E-L Au, M Sashindranath, R J Borg, O Kleifeld, R K Andrews, E E Gardiner, R L Medcalf, A L Samson

**Affiliations:** 1 Australian Centre for Blood Diseases, Department of Clinical Haematology, Monash University, Victoria 3004, Australia; 2 Department of Biochemistry, Monash University, Victoria 3800, Australia

## Abstract

Platelet activation is a frontline response to injury, not only essential for clot formation but also important for tissue repair. Indeed, the reparative influence of platelets has long been exploited therapeutically where application of platelet concentrates expedites wound recovery. Despite this, the mechanisms of platelet-triggered cytoprotection are poorly understood. Here, we show that activated platelets accumulate in the brain to exceptionally high levels following injury and release factors that potently protect neurons from apoptosis. Kinomic microarray and subsequent kinase inhibitor studies showed that platelet-based neuroprotection relies upon paracrine activation of the epidermal growth factor receptor (EGFR) and downstream DNA-dependent protein kinase (DNA-PK). This same anti-apoptotic cascade stimulated by activated platelets also provided chemo-resistance to several cancer cell types. Surprisingly, deep proteomic profiling of the platelet releasate failed to identify any known EGFR ligand, indicating that activated platelets release an atypical activator of the EGFR. This study is the first to formally associate platelet activation to EGFR/DNA-PK – an endogenous cytoprotective cascade.

Platelets are small discoid anuclear cell fragments that are key players in haemostasis – a frontline physiological response to acute tissue injury. Under basal conditions, platelets circulate at 150–400 × 10^9^ per litre and sequester a diverse array of bioactive molecules within their intracellular granules.^1^ Upon tissue injury, however, vascular disruption triggers localised platelet deposition, activation and release of their granular contents. These platelet-released molecules (PRMs) further recruit and activate platelets, resulting in a multi-cellular aggregate that restricts blood loss. In conjunction, the coagulation cascade becomes activated, leading to thrombin generation which consolidates the growing thrombus by promoting further platelet activation and by catalysing fibrin formation.

In addition to this critical role in haemostasis, platelets also participate in numerous non-haemostatic processes, including inflammation,^2^ tissue repair,^3^ angiogenesis and lymphatic development.^4^ Platelets also promote tumour cell proliferation and metastasis *via* peri-cellular adhesion and signalling.^5, 6, 7^ Such pleiotropy is attributed to the array of molecules that are released from activated platelets.

The reparative influence of platelets also has important clinical utility. For decades, ‘platelet concentrates' have been applied to sites of injury to expedite the recovery of organs such as bone,^8^ skin^9^ and tendon.^10^ The beneficial effects of platelet concentrates are thought to be due to the many growth factors that are released from activated platelets.^3^ This reasoning, however, may be considered speculative given the lack of direct experimental evidence. Furthermore, little-to-no information exists about the platelet-derived peri-cellular signals that facilitate tissue repair.

Platelets are normally restricted to the intravascular compartment. However, extravascular platelet accumulation can also occur under certain pathological conditions. One notable example of this is neurotrauma. Here, we find that exceptionally high levels of platelet activation occur in the brain after neurotrauma, thereby providing in-principle support that platelet products may influence neuronal survival. We therefore examined the influence of PRMs on injured primary cortical neurons. As neurons are post-mitotic, this approach allowed the elucidation of effects that were unrelated to proliferation. We find that PRMs selectively and potently reduce neuronal apoptosis *via* paracrine activation of the epidermal growth factor receptor (EGFR) and downstream activation of DNA-dependent protein kinase (DNA-PK) – a ubiquitous DNA repair enzyme. Strikingly, the same platelet-dependent mechanism also protects several non-neuronal human cancer cell types from chemotherapy-induced apoptosis. Thus, activated platelets trigger a potent and broad-acting paracrine signal that attenuates apoptosis.

Our findings highlight a beneficial action of platelets that likely operates within the injured brain where existing knowledge about platelets has been limited to its haemostatic role. Interestingly, we also find that this underappreciated cytoprotective action of platelets may have implications in cancer where the association between higher platelet count and poorer patient prognosis has been well established.^11, 12, 13, 14^ In addition, this newly identified anti-apoptotic mechanism should encourage rationally designed improvements to the clinical use of platelet concentrates.

## Results

**Figure 1:**
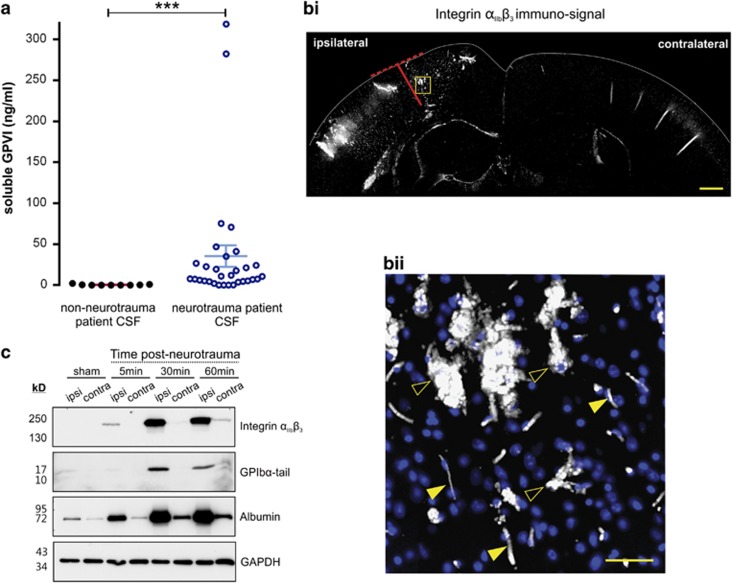
Platelets accumulate and activate in the brain after neurotrauma. (**a**) ELISA measuring soluble GPVI in the CSF of non-neurotrauma patients (*n*=9, black opened circles) and neurotrauma patients (*n*=31, blue closed circles). The mean±95% confidence intervals are indicated. ***P<0.001 by two-tailed Mann–Whitney test. (**b**) (i) Representative immunofluorescence for the platelet marker integrin *α*_IIb_*β*_3_ shows marked platelet deposition in the injured ipsilateral cortex 30 min after neurotrauma compared with the corresponding contralateral cortex. Diameter of impactor is indicated by the red discontinuous line. The depth of the impact is indicated by the continuous red line. (ii) Magnified image of an area of the ipsilateral cortex (indicated by the yellow box in i). Immunosignal of integrin *α*_IIb_*β*_3_ is depicted in white and the Hoechst 33342 signal is in blue. Closed arrowheads show intravascular platelet deposits. Opened arrowheads show large extravascular platelet deposits. (**c**) Representative immunoblot of mouse brain lysates taken from the injured ipsilateral (ipsi) and uninjured contralateral (contra) hemispheres. Integrin *α*_IIb_*β*_3_ was used as a platelet marker. The cleaved fragment of GPIb (GPIb*α*-tail) was used as a platelet activation marker. Albumin was used as an indicator of blood–brain barrier breakdown. GAPDH was used as a loading control. Scale bar: (**b**) (i) 500 *μ*m. (ii) 50 *μ*m

Understanding about platelets during acquired brain injuries largely pertains to their role in haemostasis. Given their reparative influence on other tissues, however, platelets also likely exert a non-haemostatic beneficial effect on the injured brain. We first sought to determine the extent to which PRMs were present in the brain after neurotrauma, an acquired brain injury where extreme platelet activation likely occurs. To this end, we measured soluble GPVI (sGPVI; a platelet-specific marker that is shed from activated platelets^15^), in the cerebrospinal fluid (CSF) of neurotrauma patients (Figure 1a). Whereas control patients had little-to-no sGPVI in their CSF (mean=0.45 ng/ml), sGPVI levels in the CSF of neurotrauma patients were significantly elevated (mean=35.42 ng/ml). To our knowledge, this is the first quantification of any PRM in the brain after neurotrauma. Remarkably, the level of sGPVI in the CSF of neurotrauma patients was comparable to that in plasma during disseminated intravascular coagulation, which lie between 39.9 and 72.8 ng/ml.^15^ Thus, profound intracranial release of platelet-derived molecules occurs after neurotrauma.

To establish the spatiotemporal characteristics of platelet activation/deposition after neurotrauma, we used a mouse model of neurotrauma; where a single unilateral 2 mm blunt-force impact was delivered to the cerebral cortex. After injury, mice were transcardially perfused to remove free-flowing blood and their brains subjected to immunofluorescence for the platelet-specific marker, integrin *α*_IIb_*β*_3_. As shown in Figure 1b, considerable platelet accumulation was evident within the injured ipsilateral hemisphere, but not within the uninjured contralateral hemisphere 30 min after neurotrauma. Notably, platelets were deposited not only within the initial impact site (red-coloured axes; Figure 1bi) but also at distal secondary lesion sites within the ipsilateral hemisphere where apoptotic cell death predominates. Platelet deposition within the trauma lesion was not confined to blood vessels, with numerous extravascular platelet-rich aggregates occurring within the injured brain parenchyma (Figure 1bii). Similar results were observed when immunofluorescence for the platelet-specific marker, GPIb*α*, was performed (data not shown). These studies confirm that widespread platelet activation occurs throughout the ipsilateral hemisphere after focal neurotrauma.

To establish a time course of platelet activation after injury, mice were transcardially perfused 5–60 min after neurotrauma and lysates of the ipsilateral and contralateral cortex were prepared. These lysates were then subjected to immunoblot analysis. As shown in Figure 1c, a greater extent of platelet deposition (presence of the platelet-specific integrin, *α*_IIb_*β*_3_) and platelet activation (presence of the platelet activation-specific marker, the ∼17-kDa GPIb*α* fragment) was observed in the injured ipsilateral cortex relative to the uninjured contralateral cortex. Importantly, higher levels of serum albumin were also observed in the ipsilateral cortex across all time points relative to the corresponding contralateral cortex (Figure 1c), indicating that blood–brain barrier breakdown had occurred. Hence, shortly after neurotrauma (30–60 min), pronounced platelet deposition/activation and blood–brain barrier disruption coincide within the ipsilateral cortex.

Altogether, these experiments show that overlapping blood–brain barrier breakdown and platelet deposition/activation allow PRMs to enter the injured brain parenchyma. The spatial deposition of platelets is vast and spans well beyond the initial contusion site. In addition, the magnitude of platelet deposition/activation is such that the concentration of platelet-derived sGPVI in the CSF after neurotrauma approximates that found in plasma during disseminated intravascular coagulation. Importantly, this comparison provided us with a pathophysiologically relevant concentration for using PRMs *in vitro* (PRMs were used *in vitro* at less than half the concentration to that found in the CSF of neurotrauma patients).

### PRMs attenuate neuronal apoptosis

**Figure 2:**
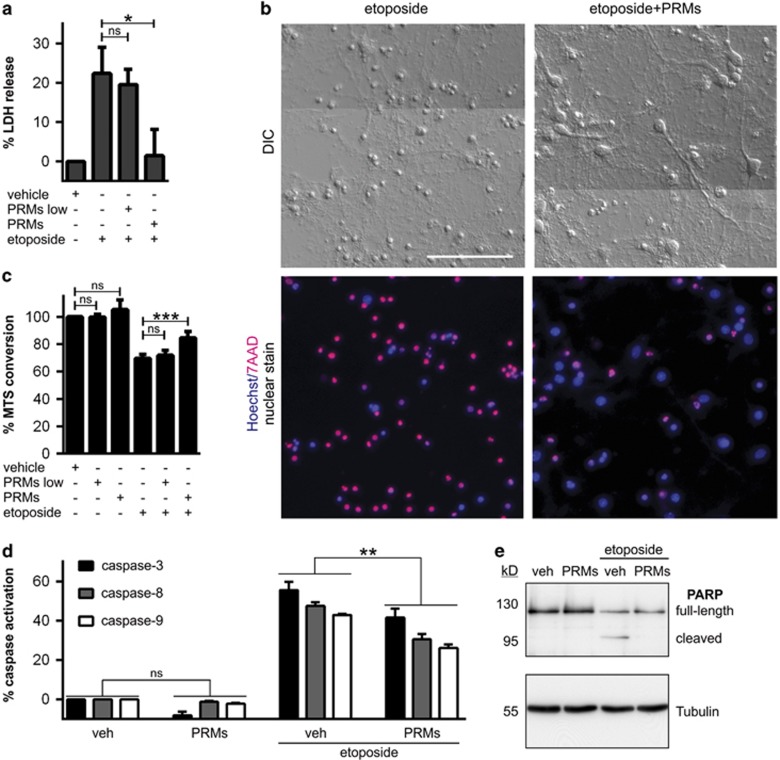
PRMs attenuate etoposide-induced toxicity in neurons. Primary mouse cortical neuronal cultures were treated with vehicle, 2 mg/l etoposide, etoposide+supernatant from unactivated platelets (PRMs low) or etoposide+supernatant from thrombin-activated platelets (PRMs). The degree of injury was assessed 24 h later by measuring plasma membrane disruption (*via* lactate dehydrogenase release (LDH); **a**; *n*=4), measuring cellular metabolism (*via* MTS conversion; **c**; *n*=5) and measuring caspase-8, -9, -3/7 activation (**d**; *n*=4). The mean with S.E.M. is indicated. *P<0.05; **P<0.01; ***P<0.001 and ‘ns' non-significant by one-way ANOVA with Newman–Keuls correction. (**b**) Representative images of neuronal cultures treated for 24 h with etoposide±PRMs, then incubated with Hoechst 33342 (coloured in blue) and 7AAD (coloured in pink). The differential interference contrast (DIC) image of the cultures is depicted in the upper row of micrographs. The epifluorescence image of the cultures is depicted in the bottom row of micrographs, with healthy cells in blue and dead cells in pink. (**e**) Representative immunoblot of lysates from neuronal cultures treated with vehicle, PRMs and etoposide±PRMs for 24 h. Immunoblots show that PRMs reduce etoposide-induced PARP cleavage. Tubulin was used as a loading control. Scale bar: (**b**) 100 *μ*m

We next investigated the influence of PRMs on neuronal injury *in vitro*. Primary mouse cortical neurons were exposed to a variety of stressors (including etoposide, L-glutamate, linsidomine and oxygen-glucose deprivation) in the presence or absence of PRMs and cellular injury assessed 24 h later. The most striking observation from these studies was that PRMs from activated platelets, but not from resting platelets, markedly reduced the magnitude of etoposide-induced injury as indicated by a PRM-mediated preservation of membrane integrity (Figures 2a and b) and cellular metabolism (Figure 2c). As etoposide is a topoisomerase-II inhibitor that induces DNA damage and subsequent apoptosis,^16^ we monitored the effect of PRMs on caspase activation. PRMs attenuated etoposide-induced activation of the upstream initiator caspases -8 and -9, reduced downstream activation of the effector caspase-3/7 (Figure 2d) and prevented caspase-dependent cleavage of PARP (Figure 2e). Hence, PRMs block an early stage apoptosis before the activation of major intrinsic/extrinsic caspases.

PRMs also protected neurons from linsidomine (Supplementary Figure S2a) – a potent nitrosative stressor. Thus, PRMs protect neurons from mechanistically distinct injuries. In contrast, PRMs failed to rescue neurons from cell death induced by L-glutamate (Supplementary Figure S2b) or oxygen-glucose deprivation (Supplementary Figure S2c). Interestingly, whereas treatment of neurons with etoposide or linsidomine triggered caspase-3 activation, L-glutamate exposure and oxygen-glucose deprivation failed to elicit any discernible caspase-3 activation (Supplementary Figures S2d and e). These findings suggest that PRMs do not protect from caspase-independent forms of cell death, but rather that PRMs specifically attenuate neuronal injury by dampening events upstream of pro-apoptotic caspase activation.

To gain further insight into the cytoprotective actions of PRMs, we assessed its influence on proteasome stress. Despite the fact that inhibition of the proteasome can activate the apoptotic cascade in both neurons and non-neuronal cells,^17, 18^ PRMs did not attenuate injury caused by MG132 treatment (Supplementary Figure S2f) or bortezomib treatment (Supplementary Figure S2g). Cellular injury caused by proteasome inhibition differs from both etoposide and linsidomine injury in that it does not principally involve DNA damage. These results therefore suggest that not only do PRMs preferentially attenuate caspase-mediated injury, but also PRMs effectively protect against apoptosis caused by DNA damage.

### Characterisation of PRM-mediated cytoprotection

**Figure 3:**
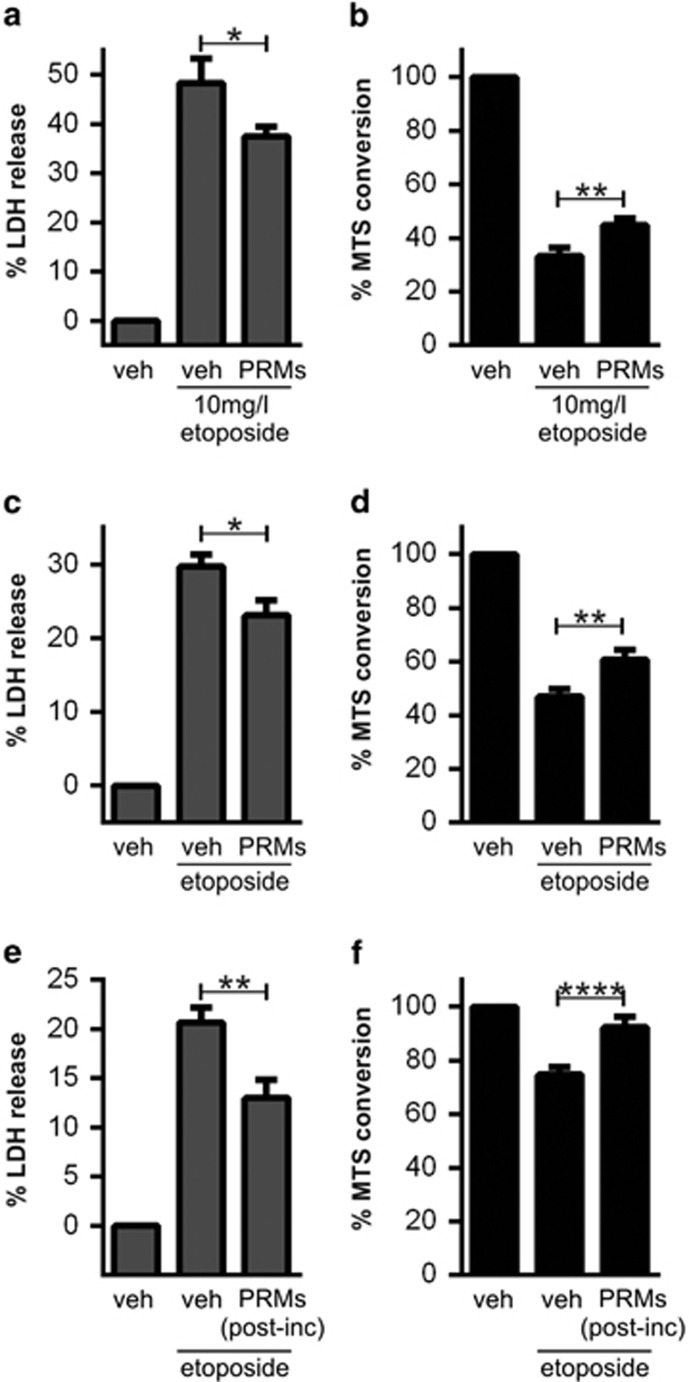
PRMs protect against high-dose etoposide and pre-initiated etoposide injury. (**a** and **b**) Primary neuronal cultures were stimulated with vehicle, 10 mg/l etoposide±PRMs. (**c** and **d**) Primary neuronal cultures were stimulated with vehicle, 2 mg/l etoposide±PRMs. (**e** and **f**) Primary neuronal cultures were stimulated with vehicle or 2 mg/l etoposide for 1 h before the addition of PRMs. The degree of injury was assessed 24 h (**a**, **b**, **e** and **f**) or 48 h (**c** and **d**) later by measuring plasma membrane disruption (*via* lactate dehydrogenase (LDH) release; **a**, *n*=4; **c**, *n*=4; **e**, *n*=3) and measuring cellular metabolism (*via* MTS conversion; **b**, *n*=5; **d**, *n*=4; **f**, *n*=7). Mean with S.E.M. is indicated. *P<0.05; **P<0.01; ****P<0.0001 and ‘ns' non-significant by one-way ANOVA with Newman–Keuls correction

As neuroprotection had not previously been directly linked to platelet activation, we further characterised the capacity of platelets to attenuate apoptosis. First, we assessed whether neuroprotection could still be elicited by *in situ* activated platelets (rather than by purified PRMs), because our protocol for preparing PRMs removes molecules <3 kDa and allows the degradation of labile platelet-derived agents (e.g., thromboxane A2). These omitted molecules, however, do not principally alter the cytoprotective effect of PRMs, as *in situ* activation of washed platelets still reduced etoposide-induced injury in neuronal cultures (*P<0.05; *n*=3; data not shown). This finding shows that the net effect of platelet activation is indeed neuroprotection. We also examined the potency of PRM-mediated neuroprotection. As shown in Figures 3a and b, PRMs still reduced neuronal injury caused by a 5-fold higher concentration of etoposide. In addition, the cytoprotective influence of PRMs was observed even after 48 h of etoposide exposure (Figures 3c and d). Hence, PRMs transmit a potent and long-lasting anti-apoptotic signal to neurons. Notably, the beneficial influence of PRMs was unrelated to cellular proliferation as no bromodeoxyuridine incorporation was observed in neuronal cultures after 24 h of PRMs treatment (data not shown). Next, to assess the therapeutic potential of PRMs, we added PRMs to neuronal cultures 1 h after the commencement of etoposide injury. Despite the fact that apoptosis had already been initiated, post-injury application of PRMs significantly attenuated etoposide-induced neuronal injury (Figures 3e and f). Hence, the capacity of PRMs to rescue neurons following the onset of apoptosis suggests that the active agent within PRMs has therapeutic potential.

**Figure 4:**
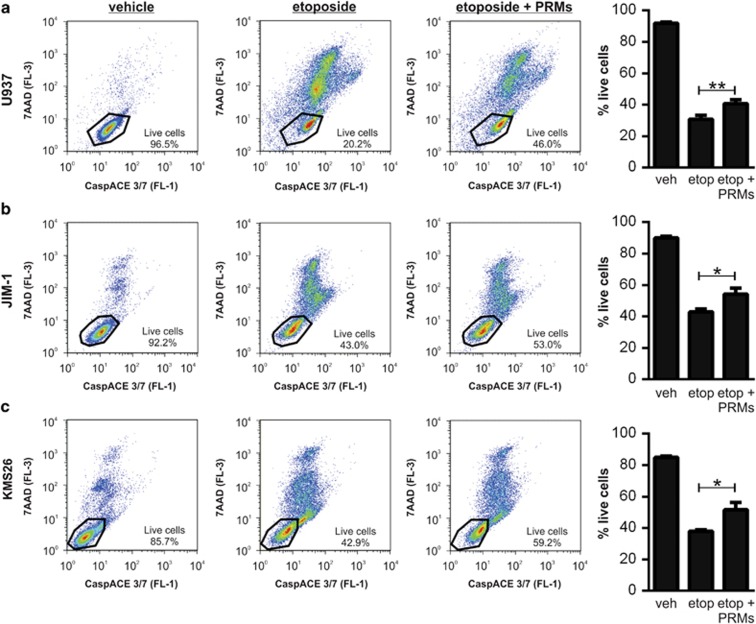
PRMs attenuate apoptosis in non-neuronal cells. The cell lines (indicated on the left) were treated with vehicle, etoposide±PRMs. The degree of injury was assessed 24 h by staining with the nuclear dye 7AAD (FL-3) and CaspACE FITC-VAD-FMK (FL-1). The three left-hand columns depict the raw data (as a scatter plot of FL-1 versus FL-3) of a single representative experiment, with the live cell gate superimposed. The right-hand column depicts the portion of live cells collated across multiple experiments showing that etoposide toxicity was reduced by PRMs in (**a**) monocytic cells U937 (*n*=8), (**b**) plasma cells JIM-1 (*n*=3) and (**c**) lymphocyte-like cells KMS26 (*n*=3). Mean with S.E.M. is indicated. *P<0.05, **P<0.01 and ‘ns' is non-significant by one-way ANOVA with Newman–Keuls correction

Finally, we investigated whether PRMs could also attenuate apoptosis in other cell types. Given the well-recognised contribution of platelets to cancer progression,^6^ we assessed the ability of PRMs to inhibit etoposide-induced apoptosis in a range of human cancer cell lines. PRMs reduced the extent of etoposide-induced cell death in monocytic lymphoma cells (U937, Figure 4a), myelocytic plasma cells (JIM-1, Figure 4b) and myelocytic lymphocytes (KMS26, Figure 4c). These findings broaden the importance of platelet-mediated cytoprotection and are significant as etoposide is a chemotherapeutic used to treat malignancies, including small cell lung carcinoma, germ-line cancers, sarcoma, lymphoma and leukaemia.^19^

### Identifying signalling pathways that underlie PRM-mediated cytoprotection

**Figure 5:**
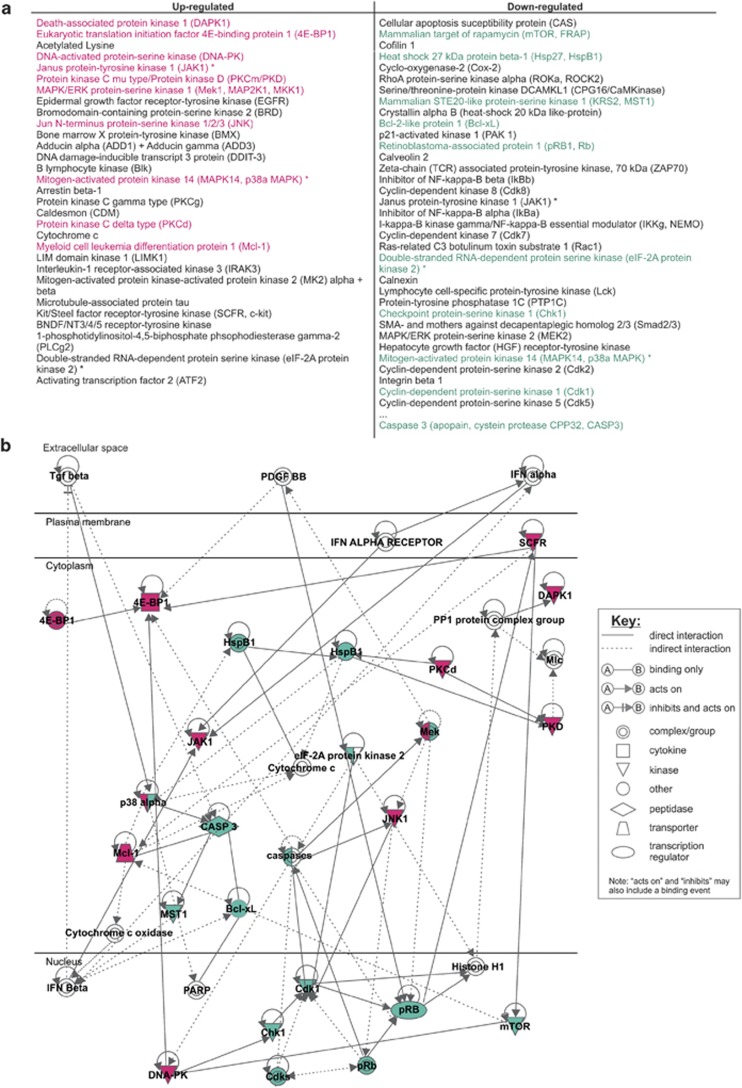
Kinomic screening and prediction of the signalling events that underlie PRM-mediated cytoprotection. (**a**) Neuronal cultures 30 min and 3 h after etoposide±PRMs stimulation were lysed and subject to kinomic microarray analysis yielding a short list of signalling proteins significantly altered (red indicates a >1.2 × upregulated event and green indicates a >0.55 × downregulated event) by PRMs following etoposide injury. (**b**) The short-listed signalling proteins (Supplementary Table 1) were subjected to Ingenuity Pathway Analysis (IPA) to yield six canonical signalling pathways (data not shown) for PRM-mediated cytoprotection. Signalling proteins that showed interplay with caspase or other apoptotic processes were manually merged (across the predicted canonical signalling pathways) to generate a single predicted signalling pathway that underlies PRM-mediated cytoprotection. Red symbols represent upregulated events. Green symbols represent downregulated events. Subsequent kinase inhibitor studies based on this putative signalling pathway highlighted DNA-PK as a key kinase effector of PRM-mediated cytoprotection

We next wanted to identify the paracrine signalling pathway responsible for PRM-mediated cytoprotection. We first tested for kinase dependency using staurosporine (a pan-kinase inhibitor that triggers DNA damage and neuronal apoptosis), and found that PRMs failed to alter the extent of staurosporine-induced neuronal injury (Supplementary Figure S3). This finding suggests that the neuroprotective action of PRMs relies upon kinase activity. To identify which kinases are activated by PRMs, we performed a kinomic screen whereby neuronal cultures were injured with etoposide in the presence/absence of PRMs. Lysates were harvested after 30 and 180 min of treatment and then subjected to Kinexus antibody microarray (which utilises ∼500 pan- and ∼340 phospho-specific antibodies). The complete microarray data sets are presented in Supplementary Table 1. Pair-wise comparison and collation of the microarray data across both time points produced a high-confidence list of signalling proteins that were differentially regulated by PRMs (Figure 5a). Importantly, the kinomic screen showed that PRMs caused a 44% reduction in caspase-3 function, verifying the integrity of the initial cell culture experiment (Supplementary Table 1).

To offer system-wide insight, the kinomic data sets were subjected to Ingenuity Pathway Analysis (Redwood City, CA, USA) where the analyst (from the Australian Proteome Analysis Facility) was blinded to both the experimental design and the overall project hypothesis. Five of the top six canonical pathways identified by Ingenuity Pathway Analysis indicated a critical PRM-mediated impact on apoptosis and predicted a reduction in caspase function (data not shown). These top five pathways were then manually collated into a single signalling pathway that putatively underlies PRM-mediated cytoprotection (Figure 5b).

### PRMs inhibit apoptosis *via* the preservation of DNA-PK activity

**Figure 6:**
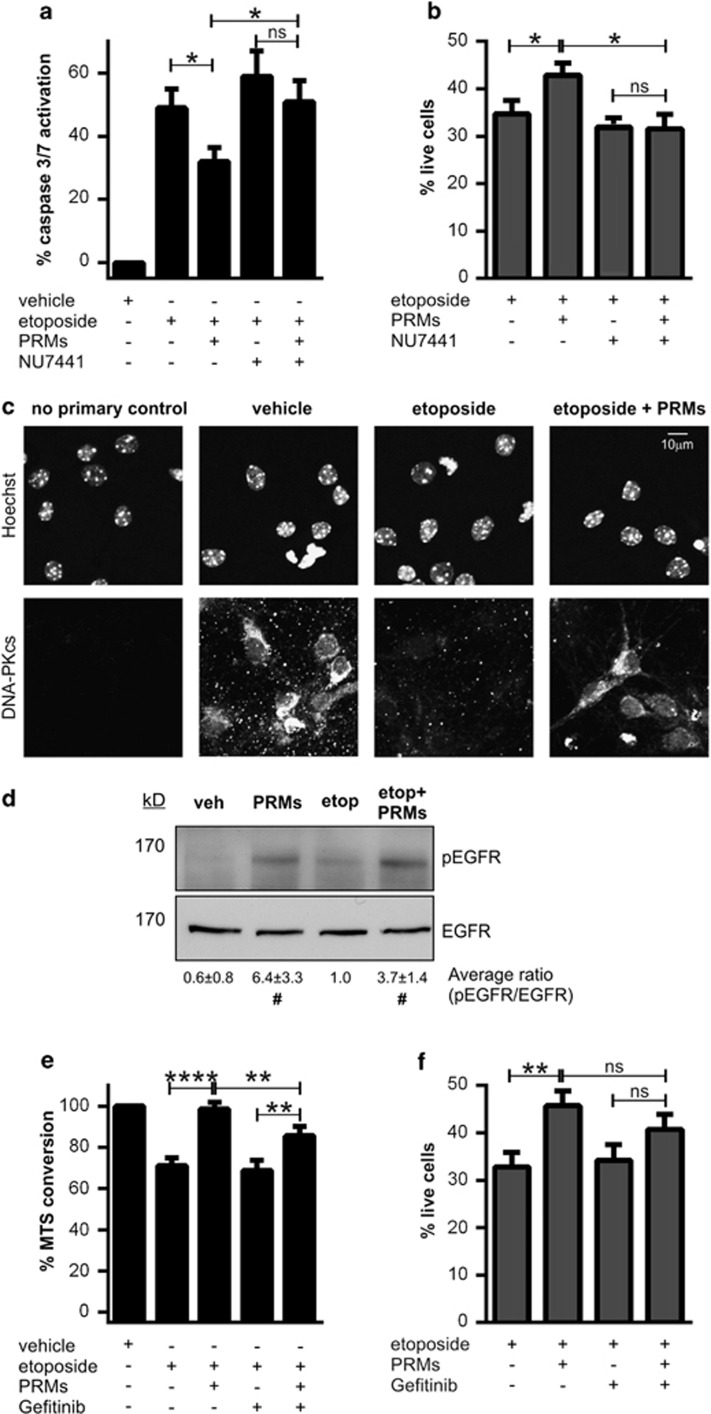
PRM-mediated cytoprotection is *via* EGFR and downstream DNA-PK activation. Neuronal cultures (**a**; *n*=5) or U937 cells (**b**; *n*=4) were stimulated with vehicle or 1–2 mg/l etoposide±PRMs±10 nM NU7441 (a DNA-PK-specific inhibitor). The degree of injury was assessed 24 h later by measuring caspase-3/7 activation (**a**) or cell viability (**b**; % negatively staining for 7AAD and CaspACE FITC-VAD-FMK). (**c**) Representative images of neuronal cultures stimulated with vehicle, 2 mg/l etoposide or 2 mg/l etoposide+PRMs. Cultures were fixed after 1 h and subjected to immunofluorescence for the DNA-PK catalytic subunit (DNA-PKcs). The top micrographs depict the Hoechst 33342 nuclear counterstain and the bottom micrographs depict the corresponding DNA-PKcs immunosignal. (**d**) Representative immunoblot of neuronal cultures stimulated with 2 mg/l etoposide±PRMs. Cultures were lysed 30 min post stimulation and subjected to immunoblot analyses for total and phosphorylated EGFR (pEGFR). Subtext is the densitometry analysis comparing the average ratio of pEGFR/EGFR (mean±S.D.; *n*=4; ^#^*P*<0.05 between groups with/without PRMs using unpaired *t*-test with Welsh correction). Cultures treated in the presence of PRMs have significantly elevated pEGFR/EGFR levels compared with cultures treated in the absence of PRMs. Neuronal cultures (**e**) or U937 cells (**f**) were stimulated with vehicle or 1–2 mg/ml etoposide±PRMs±Gefitinib (an EGFR-specific inhibitor; 0.4 *μ*M for neuronal cultures; 10 *μ*M for U937s). The degree of injury was assessed 24 h later by measuring cellular metabolism (*via* MTS conversion; **e**; *n*=4) or cell viability (**f**; *n*=4; % negatively stained for 7AAD and CaspACE FITC-VAD-FMK). *P<0.05, **P<0.01, ***P<0.001 and ‘ns' is non-significant by one-way ANOVA with Newman–Keuls correction. ^#^*P*<0.05 by unpaired *t*-test with Welsh correction. Scale bar: (**c**) 10 *μ*m

On the basis of the putative signalling pathway outlined in Figure 5b, selective pharmacological inhibitors of p38*α*, JNK1, JAK1, DAPK1 (data not shown) and DNA-PK were sequentially tested for their capacity to abrogate PRM-mediated cytoprotection. Of the tested inhibitors, only the DNA-PK-selective compound, NU7441, attenuated PRM-mediated cytoprotection in both mouse cortical neurons and human U937 monocytes (Figures 6a and b, respectively). Hence, paracrine activation of DNA-PK mediates the cytoprotective influence of PRMs.

DNA-PK is a ubiquitous enzyme responsible for the repair of double-stranded DNA breaks.^20^ Interestingly, our kinomic screen indicated that DNA-PK levels were 317% higher in neuronal cultures following etoposide+PRM treatment, relative to cultures treated with etoposide alone (Figure 5a; Supplementary Table 1). In support of these findings, immunofluorescence studies showed that 1 h of etoposide treatment dramatically reduced DNA-PK levels, whereas co-treatment with PRMs fully restored DNA-PK to basal levels (Figure 6c). Hence, our data suggest that an immediate-early degradation of DNA repair machinery contributes to etoposide toxicity and that PRMs protect by preserving DNA-PK-mediated DNA repair.

### Platelets inhibit apoptosis *via* atypical activation of the EGFR

While DNA-PK is a recognised cytoprotectant,^21, 22^ the receptors that trigger its activation are poorly understood. The only well-established receptor that promotes DNA-PK activation is the EGFR.^23, 24^ Notably, our kinomic screen showed that PRMs, in addition to increasing DNA-PK levels, also triggered a concomitant 192% increase in phospho-EGFR levels (Figure 5a; Supplementary Table 1). We therefore hypothesised that PRMs protect *via* activation of the ‘EGFR→DNA-PK' cascade. To test this hypothesis, we first confirmed that PRMs did indeed increase activation of EGFR (Figure 6d). Next, we blocked the EGFR using the anti-cancer compound, Gefitinib – a small-molecule inhibitor of the EGFR.^25, 26^ As shown in Figures 6e and f, Gefitinib significantly diminished PRM-mediated cytoprotection against etoposide exposure in both mouse neurons and human U937 monocytes. These findings suggest that the cytoprotective signal transduced by PRMs is mediated by EGFR activation.

The EGFR has a well-defined repertoire of extracellular ligands, namely: EGF, Transforming Growth Factor-*α*, heparin-binding-EGF, Amphiregulin, Betacellulin, Epigen, Epiregulin and Neuregulins 1–6.^25^ We performed deep proteomic profiling to determine whether any EGFR ligands were present in our preparations of PRMs. In all, 394 proteins were identified in PRMs, of which 266 were confidently identified by two-or-more peptides (Supplementary Table 2). However, no EGFR ligand was confidently identified in our preparations of PRMs. Similarly, no other prior characterisation of the platelet releasome has detected the presence of a recognised EGFR ligand (Wijten *et al*^1^ and references therein). While no recognised EGFR ligand was detected in PRMs, proteins containing an ‘EGF-like domain' represented the most abundant class of proteins in PRMs (Supplementary Table 2). Hence, activated platelets release a novel ligand of the EGFR that, in turn, promotes cell survival during genotoxic insult *via* the maintenance of DNA-PK-dependent DNA repair.

## Discussion

**Figure 7:**
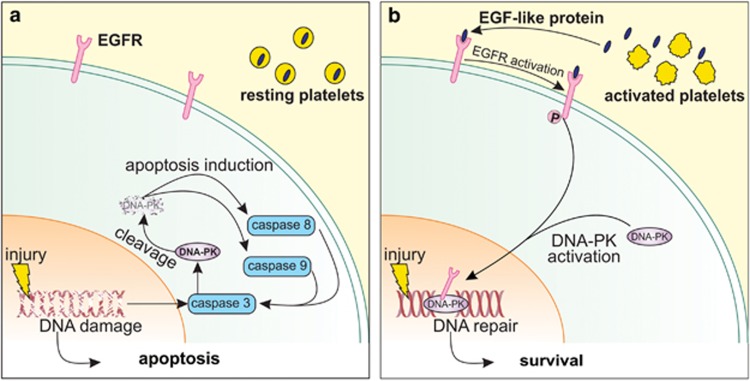
Working model of PRM-mediated cytoprotection against etoposide injury. (**a**) Etoposide injury in the absence of platelet activation allows etoposide to injure cells by inhibiting DNA-topoisomerase II and inducing double-stranded DNA damage. Initial caspase activation following DNA damage leads to DNA-PK degradation, thereby preventing DNA repair and promoting irreversible initiation of the caspase-8 (extrinsic) and caspase-9 (intrinsic) arms of apoptosis. (**b**) Etoposide injury in the presence of platelet activation does not eventuate in apoptosis. This is because activated platelets release an EGF-like protein (blue) that binds to and promotes phospho-activation of the EGFR. The phospho-activated EGFR translocates to the nucleus and forms a complex with DNA-PK. As the EGFR:DNA-PK complex is resistant to caspase-mediated degradation, any etoposide-induced DNA damage is amenable to repair and thus leads to cell survival

Platelet activation is a primary response to tissue injury. While thrombus formation is the best characterised consequence of platelet activation, proper initiation of inflammation, angiogenesis, proliferation and tissue repair also depends upon platelet activation. Recent research suggests that peri-cellular protection may be another consequence of platelet activation.^7, 27, 28^ Our study uniquely shows that activated platelets release an atypical agonist of the EGFR, which in turn, transduces a paracrine signal that potently attenuates apoptosis *via* the preservation of DNA-PK-dependent DNA repair. Figure 7 presents our working model of platelet-based cytoprotection, whereby activated platelets release an EGF-like protein that triggers peri-cellular activation and nuclear translocation of the EGFR, leading to EGFR:DNA-PK complex formation, the preservation of DNA-PK levels and the promotion of DNA repair. In support of this mechanism, prior studies have shown that EGFR:DNA-PK complex maintains DNA repair^23^ presumably *via* blockade of caspase-mediated degradation of DNA-PK during genotoxic stress.^29^ Hence, a ‘therapeutic window' likely exists for DNA-PK-mediated DNA repair during genotoxic stress. We theorise that injury-induced platelet activation aligns with this ‘therapeutic window' and helps to prevent the irreversible activation of intrinsic and extrinsic apoptosis (Figure 7).

Although our data indicate that EGFR-DNA-PK signalling is a major mediator of platelet-triggered cytoprotection, they do not exclude the contribution of other pro-survival signalling cascades. Indeed, the predicted canonical pathway outlined in Figure 5b stipulates that other kinases including p38*α*, JNK1, JAK1, 4E-BP1 and DAPK1 are activated by PRMs. Studies should now address whether PRM-mediated activation of these additional kinases subserves other non-haemostatic influences of platelets.

The anti-apoptotic factor(s) released from activated platelets has several favourable characteristics. First, PRMs offer protection to a range of cell types (Figure 4) and, importantly, can be used to rescue injured cells after apoptosis has been initiated (Figures 3e and f). The potency of platelet-based anti-apoptosis is also noteworthy, with PRMs often completely protecting neurons from injury (Figures 2a and 6e; Supplementary Figure S2a). This potency may be due to the fact that PRMs interfere with a very early step in apoptotic induction; with PRMs diminishing activation of initiator caspases (Figure 2d), preventing an immediate-early drop in DNA-PK levels after etoposide treatment (Figure 6c), and attenuating etoposide-induced phosphatidylserine exposure in U937 cells (data not shown). It is also notable that PRMs maintained their protective action after heating (>50 ^o^C; data not shown) and that the protective agent lacked pro-thrombotic activity when purified from other PRM constituents (data not shown). Thus, the anti-apoptotic factor in PRMs harbours desirable traits that encourage its future identification and development as a potential therapeutic.

Several points pertaining to the preparation and use of PRMs warrant mention. First, PRMs were prepared by pooling the supernatants from washed activated human platelets across ≥4 independent donors. Our preparations of PRMs were also of high purity, significantly overlapping with the other published in-depth characterisation of the platelet releasome (Wijten *et al.*^1^ and references therein). In addition, numerous independent batches of PRMs were used throughout this study, with each batch offering potent cytoprotection. Hence, our study denotes that a potent anti-apoptotic agent is constitutively expressed and reproducibly released from activated human platelets.

### Platelets, EGFR and DNA-PK in the context of brain injury

Only recently have studies begun to define the non-haemostatic roles of platelets in the brain. For example, recent studies show that platelets drive neuroinflammation during experimental stroke and multiple sclerosis.^30, 31^ However, discrepant findings exist with respect to the capacity of platelets to influence neurotoxicity. One research group has shown that activated platelets are toxic to the central nervous system,^32, 33^ whereas another study shows that intracranial administration of platelet lysates (rather than PRMs) reduces post-stroke lesion volume and neurological deficit in spontaneously hypertensive rats.^34^ Hayon *et al.*^34^ attributed the beneficial effect of platelet lysates to an increase in angiogenesis and proliferation and also concluded that a platelet-based promotion of cell survival likely contributed to the dramatic improvement in post-stroke injury. Our observations that platelets offer potent neuroprotection complement and provide an underlying molecular mechanism for the findings of Hayon *et al.* ^34^ Following stroke and neurotrauma, for example, such a platelet-based mechanism might delay apoptosis, affording time for the restoration of tissue homeostasis. Indeed, a platelet-based neuroprotective mechanism capitalises on the fact that platelets are frontline responders to injury capable of releasing anti-apoptotic effectors in a spatially and temporally appropriate manner.

In comparison with the emerging neural roles of platelets, it is well established that EGFR signalling can protect the brain from injury.^35, 36, 37, 38, 39^ Similarly, DNA-PK is a recognised neuroprotectant, with studies showing that DNA-PK^−/−^ neurons are hypersensitive to injury.^22, 40^ Surprisingly, no prior study has established whether EGFR-mediated activation of DNA-PK is a protective pathway that operates during brain injury. Thus, a PRM that activates the EGFR-DNA-PK cascade (our present study) warrants further investigation.

### Platelets, EGFR and DNA-PK in the context of cancer

It is well accepted that activated platelets deleteriously contributes to cancer aetiology.^6, 41^ It is thought that activated platelets ensheath circulating cancer cells, allowing them to evade immune detection, protecting them from vascular shear forces and helping vessel wall attachment and metastasis.^41, 42, 43^ In addition, activated platelets release paracrine factors that promote cancer cell proliferation,^44^ chemo-resistance^7^ and angiogenesis.^45, 46^ Hence, platelet count is commonly used as a prognostic factor whereby patients with high platelet counts have reduced survival rates in a range of tumour types including cervical^47^ and lung cancer.^48^ Our data extend these observations and show that activated platelets increase the survival of cancer cells exposed to etoposide – a widely used chemotherapeutic. Therefore, we predict that blockade of platelet-based cytoprotection, in conjunction with radio/chemotherapy may enhance the efficacy of cancer treatments.

Interestingly, ectopic activation of the EGFR-DNA-PK cascade has also been closely linked to tumourigenesis and radio/chemo-resistance.^49, 50, 51, 52, 53^ Indeed, as a high proportion of malignancies feature hyperactivation/mutation of EGFR, the EGFR is a mainstay of anti-cancer research.^25, 54, 55, 56^ Similarly, DNA-PK-selective antagonists, including NU7441, are promising new anti-cancer drugs.^51, 57^ In this context, discovery of platelets as a potent, atypical and injury-specific activator of EGFR-DNA-PK signalling is of explicit relevance to the field of cancer. Clinical trial records should now be stratified to determine whether the efficacy of EGFR-inhibitory regimes correlates with platelet counts in cancer patients.

### Concluding remarks

The widespread medical use of platelet concentrates to facilitate tissue repair led us to investigate whether pro-survival factors are released by activated platelets. We find that molecules released from platelets that are yet to be identified, potently protect a variety of cell types from apoptosis by transactivation of the EGFR and stimulation of downstream DNA-PK-mediated DNA repair. Our study is the first to connect platelets, frontline responders to tissue injury, to the highly studied EGFR-DNA-PK cascade. While this connection uncovers new avenues for neuroprotection, it also provides a plausible mechanistic basis for the correlative actions of platelets, EGFR and DNA-PK during cancer. Future research should now address platelet-based cytoprotection during neurotrauma, cancer, the clinical use of platelet concentrates, and indeed, any situation where large-scale platelet activation and apoptosis coincide.

## Materials and Methods

### Materials

All reagents were from Life Technologies (Carlsbad, CA, USA) unless otherwise indicated. Etoposide, L-glutamate, staurosporine, MG132 and linsidomine (3-morpholinosydnonimine hydrochloride; SIN-1) were form Sigma (St. Louis, MO, USA). NU7441 was from Tocris Bioscience (Bristol, UK). Gefitinib was from Cayman Chemical (Ann Arbor, MI, USA).

### Acquisition of human CSF

CSF collection was performed in accordance with the Australian National Health and Medical Research Council's (NHMRC) National Statement on Ethical Conduct in Research Involving Humans and was approved by the Alfred Hospital Human Ethics Committee. Thirty-one patients (7 female and 24 males; aged between 15 and 73 years old; mean age of 32.1 years) were recruited *via* the Trauma Service of the Alfred Hospital, Melbourne, with delayed informed consent obtained from the next of kin. Patients who suffered from severe neurotrauma determined by a post-resuscitation and pre-intubation Glasgow Coma Scale Score (GCS) ≤9 and the requirement of an extraventricular drain for monitoring intracranial pressure (ICP) were included in the study. CSF was drained from patients when the ICP was greater than 20 mm Hg and collected in bags in cooled 4 °C containers over 24 h. CSF samples collected within 4 days of admission were centrifuged at 2000 r.c.f. for 15 min at 4 °C, and stored at −80 °C until needed. Exclusion criteria included pregnancy, known history of neurodegenerative diseases, HIV and other chronic infection or inflammatory diseases, history of previous neurotrauma and age <15 years. Patients with focal brain injury (classified as the presence of either an evacuated lesion or a non-evacuated high- or mixed-density mass lesion greater than 25 ml in volume) or/and diffuse brain injury (defined by the presence of Grade I to IV diffuse injury based on the degree of compression of cisterns and the occurrence of a midline shift) were included in the study.

### Animals

All animal procedures adhered to the NHMRC Code of Practice for the Care and Use of Animals for Experimental Purposes in Australia and were approved by the institutional Animal Ethics Committee. All experiments used C57Black/6 mice. Experimental neurotrauma was performed on 8- to 12-week-old male C57Black/6 mice. Neuronal cultures were prepared from the embryos of 4- to 12 week-old pregnant mice.

### Measuring sGPVI in CSF

The measurement of sGPVI was performed as in Al-Tamimi *et al.*^58^ with minor modifications. CSF samples were analysed in duplicate. CSF from neurotrauma patients was diluted 1:5 in artificial CSF (0.3 M NaCl, 6 mM KCl, 2.8 mM CaCl_2_.2 H_2_O, 1.6 mM MgCl_2_.6 H_2_O, 1.6 mM Na_2_HPO_4_.7 H_2_O, 0.4 mM NaH_2_PO_4_.7 H_2_O) to a final volume of 100 *μ*l. Each ELISA plate contained standards of recombinant GPVI ectodomain (0–100 ng/ml final concentration) serially diluted in artificial CSF.

### Experimental neurotrauma

Neurotrauma on mice was induced using the controlled cortical impact model as in Samson *et al.*^59^ In brief, a burr hole was drilled over the left parietal cortex of anaesthetised mice and a single blunt impact (5 m/s velocity; 2 mm impact depth; 150 ms dwell time) was delivered by a controlled impactor device. Mice that were designated as ‘sham' mice were subjected only to the craniotomy procedure after anaesthesia.

### Extraction and homogenisation brain tissue

Mice were transcardially perfused at fixed time points after neurotrauma with 30 ml PBS (pH 7.4) containing 10 mM EDTA. Brains were removed and 2 mm coronal sections cut using a Mouse Brain Blocker (Kopf, Tujunga, CA, USA). The cortex was then dissected from these sections, weighed and homogenised to a final concentration of 150 mg of tissue/ml in PBS+1% Triton X-100, containing 10 *μ*M GM6001 as well as 1 × phosphatase and protease inhibitor cocktail tablets (Roche, Mannheim, Germany).

### Immunohistochemistry

PBS/EDTA-perfused brains were removed and formaldehyde fixed (4 °C overnight). Brains were then dehydrated overnight and paraffin embedded using an automatic tissue processor. In all, 6 *μ*m tissue sections were cut and processed for immunohistochemistry. Sections were stained with 1:100 rat anti-mouse integrin *α*_IIb_*β*_3_ (BD Biosciences, San Jose, CA, USA) or 1:100 rat anti-mouse GPIb*α* (Emfret Analytics, Eibelstadt, Germany) overnight at 4 °C. Sections were washed and incubated with a fluorescent-conjugated secondary antibody and for 2 h at room temperature, followed by 5 mg/l Hoechst 33342 for 5 min. Slides were washed and cover-slipped in fluorescence mounting media (DAKO, Glostrup, Denmark). Images were captured on the Zeiss Cell Observer system (microscope: Axio Observer.Z1, Jena, Germany; objective#1 (EC Plan-Neofluar, × 40 magnification, 0.75 numerical aperture, air immersion) or objective #2 (Plan-Apochromat 63 × magnification, 1.40 numerical aperture, oil immersion); sequential excitation and emission filters for 4',6-diamidino-2-phenylindole, fluorescein, and Texas Red fluorophores; camera: AxioCamMR3; acquisition software: AxioVision 4.8, Jena, Germany). Images were processed using the ImageJ v1.47q software (National Institutes of Health, Bethesda, MD, USA).

### Immunoblot analysis

Samples were boiled in SDS-loading buffer with dithiothreitol (DTT), subjected to sodium dodecyl sulphate-polyacrylamide gel electrophoresis and transferred onto polyvinylidene fluoride membranes. Primary antibodies were rabbit anti-human GPIb*α*-tail,^58^ mouse anti-human GPIb*α* (WM23, DAKO), rat anti-mouse integrin *α*_IIb_*β*_3_ (BD Biosciences), rabbit anti-human GPVI (12A5;Al-Tamimi *et al.*^58^), rabbit anti-polyclonal cyclophilin A (Abcam, Cambridge, UK), rabbit anti-polyclonal ADP-ribose polymerase (PARP, Santa Cruz Biotechnology, Dallas, TX, USA), goat anti-human albumin (Bethyl Laboratories Inc., Montgomery, TX, USA), mouse anti-GAPDH (Merck Millipore, Billerica, MA, USA), mouse anti-*β* tubulin (Sigma), rabbit anti-EGFR (D38B1, Cell Signaling, Danvers, MA, USA), rabbit anti-phospho-EGFR Tyr1173 (53A5, Cell Signaling). Appropriate HRP-conjugated secondary antibodies were used and signals revealed by chemiluminescence (Thermo Scientific, Bremen, Germany).

### Preparation of platelet-rich plasma and washed platelets

All human blood collection was in accordance with the ethical standards of the Monash University Standing Committee for Ethics involving Research into Humans and with the Helsinki Declaration of 1975. Human plasma from healthy donors was collected and platelets were washed as in Kulkarni *et al.*^60^ In brief, human whole blood was collected into acid citrate dextrose at a ratio of 6:1 (blood:acid). In all, 20 U/ml Clexane was added and incubated for 15 min at 37 °C. All subsequent procedures were performed at 37 °C. Separation of platelet-rich plasma and red blood cells was achieved by centrifugation (300 r.c.f., 16 min). The platelet-rich plasma was collected and left to rest for 10 min before centrifugation (1700 r.c.f., 7 min). The platelet pellet was then resuspended in platelet wash buffer (4.3 mM K_2_HPO_4_, 4.3 mM Na_2_HPO_4_, 24.3 mM NaH_2_PO_4_, 113 mM NaCl, 5.5 mM D-glucose, and 10 mM theophylline, pH 6.5). Platelets were allowed to rest for 30 min before use.

### Preparation of PRMs

Two different preparations of PRMs were used for this study: (1) directly from whole blood as described above or (2) from expired platelet bags obtained through the Alfred Hospital Blood Services. The cytoprotective effect of PRMs was evident irrespective of the preparation methodology. Platelets (from either procedure) were centrifuged (800 r.c.f., 7 min, 37 °C) and resuspended in modified Tyrode's buffer (10 mM HEPES, 12 mM NaHCO_3_, 13.7 mM NaCl, 2.7 mM KCl, and 5.5 mM D-glucose, pH 7.3) containing 1 mM CaCl_2_. Platelets were allowed to rest for 30 min before activated *via* the addition of 1 U/ml bovine thrombin (Merck Millipore). The activated platelet suspension was placed on a rocker (10 min, 37 °C) to ensure maximal platelet activation. As an ‘unactivated PRMs' control, supernatants were collected from platelets resuspended in Tyrode's buffer with 1 mM CaCl_2_, 20 mM theophylline, 20 U/L apyrase and 5 mM EGTA. The vehicle control for PRMs was modified Tyrode's buffer with 1 mM CaCl_2_, 1 U/ml thrombin and DMSO (equivalent in volume and concentration to the addition of corresponding compounds). Flow cytometry for *P*-selectin and fibrinogen exposure was performed to confirm the activated/resting status of platelets (FACs Calibur; Becton Dickinson, Franklin Lakes, NJ, USA; Supplementary Figure S1a). The platelet suspensions were then centrifuged (800 r.c.f., 7 min) and supernatants collected. Supernatants were pooled across 4–15 donors and concentrated 15-fold using a 3-kDa molecular weight cutoff Amicon ultrafiltration device (Merck Millipore). The ultrafiltration of PRMs was validated by immunoblot (Supplementary Figure S1B).

### Primary neuronal cultures

Primary cortical neuronal cultures were prepared as in Samson *et al.*^59^ Cultures were seeded into 48-well plates at a concentration of 112 500 cells per well. All experiments were conducted on DIV7. Before experimentation, media was replaced with phenol-red free neurobasal media (NBM) containing 2% antioxidant-free B27, 0.5 mM L-glutamine, 50 U/ml penicillin/streptomycin (P/S). Cultures were challenged with 2–20 mg/l etoposide, 10 μM L-glutamate, 60 *μ*M 3-morpholinosydnonimine hydrochloride (linsidomine, SIN-1), 500 nM MG132, 200 nM bortezomib, 100 nM staurosporine or oxygen-glucose deprivation (OGD). For OGD, media was replaced with glucose-free NBM containing antioxidant-free B27, 0.5 mM L-glutamine, 50 U/ml P/S and cultures sealed in a modular incubator (Billups-Rothenburg Inc., Del Mar, CA, USA) filled with 95% nitrogen and 5% carbon dioxide for 4 h at 37 °C. Media was then replaced with NBM and cultures maintained under humidified 5% CO_2_, 8% O_2_ conditions for 20 h. PRMs were added at a dose equivalent to that released by 6 × 10^8^ platelets/ml. All measurements were taken 24 h after the commencement of injury unless otherwise indicated in the corresponding legend.

### Cancer cell lines

Cancer cell lines (U937, JIM-1 and KMS26) were maintained in RPMI supplemented with 10% (v/v) heat-inactivated fetal calf serum, 2 mM L-glutamine, 50 U/ml P/S under humidified 5% CO_2_. Before experimentation, cells were seeded at a density of 3.5–5.0 × 10^5^ cells/ml. Cultures were treated with etoposide (1 mg/l for U937; 2 mg/l for JIM-1 and KMS26)±PRMs (to a dose equivalent to that released by 12 × 10^8^ platelets/ml). All measurements were taken 24 h after the commencement of injury, whereby cells were centrifuged, washed and resuspended in 100 *μ*L PBS containing 2.5–5 *μ*M CaspACE FITC-VAD-FMK (Promega, Madison, WI, USA) and 5–10 mg/l 7AAD. Cells were incubated for 20 min, washed and resuspended in 150 *μ*l of PBS before analysis *via* by flow cytometry (BD FACS Calibur; 25  000 events per sample). Data were analysed using the Flowjo v7.6.5 software (Tree Star Inc., Ashland, OR, USA).

### CytoTox96 non-radioactive cytotoxicity (LDH) assay, CellTitre 96 aqueous non-radioactive cell proliferation (MTS) assay, caspase 8-, caspase 9- and caspase 3/7-Glo assays

All assays were from Promega and performed according to the manufacturer's instructions.

### Cytochemistry

Primary neuronal cultures were seeded at 112  500 cells/well in μ-well plates (Ibidi, Munich, Germany), then maintained and stimulated as described above. After 24 h, cultures were PBS washed and incubated with 300 *μ*l PBS containing 5 mg/l Hoechst 33342 and 10 mg/l 7AAD dye for 30 min. Cells were imaged using the Zeiss Cell Observer system as described above. Images were processed using the ImageJ v1.47q software.

### Immunofluorescence of DNA-PK

Immunofluorescence staining was performed as in Samson *et al.*^59^ with the minor modification that cells were blocked with goat serum, stained with a 1:100 dilution of rabbit-anti-human DNA-PK antibody (Abcam) and counterstained with 10 mg/l Hoechst 33342. Cells were imaged on a Nikon A1r-si resonant scanning confocal system (microscope: Nikon Ti, Tokyo, Japan; objective: Apo LWD, × 40 magnification, 1.15 numerical aperture, water immersion; sequential excitation: 405 nm and 546 nm laser lines; emission filters: 450/50 nm and 595/50 nm; acquisition software, NIS Elements Advanced Research, Tokyo, Japan). Images were processed using the ImageJ v1.47q software.

### Cell homogenisation

Cell homogenisation was performed as in Samson *et al.*^59^

### Phospho-protein microarray

Neuronal cultures were treated with etoposide±PRMs. At *t*=30 and *t*=180 min post treatment, homogenates were prepared and sent to Kinexus Bioinformatics (Vancouver, BC, Canada) for blinded kinomic analysis using the KAM-1.2 chip equipped with ∼500 pan-specific and ∼300 phospho-site specific antibodies.

### Mass spectrometry of PRMs

In all, 30 *μ*g of purified PRMs was diluted in 1.7 g/l ammonium bicarbonate (Sigma) and 1.7 g/l Tris(2-carboxyethyl)phosphine hydrochloride (Sigma) and incubated for 5 min at 95 ^o^C. The sample was cooled to room temperature and alkylated with 1.8 g/l iodoacetamide (Sigma) for 20 min in the dark. The samples were then digested with trypsin (Promega, Sequencing Grade) at 1:100 w/w for 3 h at 37 °C. In all, 2 *μ*l of formic acid (Sigma) was added to stop proteolysis and the resulting peptides analysed by LC-MS/MS using the Q Exactive mass spectrometer (Thermo Scientific) coupled online with an UltiMate 3000 Rapid Separation LC (RSLC) nano HPLC system (Thermo Scientific). Samples were loaded on a 100 *μ*m, 2 cm nanoviper pepmap100 trap column (Thermo Scientific) in 2% acetonitrile, 0.1% formic acid at a flow rate of 15 *μ*l/min. Peptides were eluted and separated at a flow rate of 300 *μ*l/min on Thermo RSLC nanocolumn 75 *μ*m × 50 cm, pepmap100 C18, 3 *μ*m 100 Å pore size, with a linear acetonitrile gradient from 10 to 26% in 0.1% formic acid for 55 min followed by a linear increase to 34% acetonitrile in 0.1% formic acid over 5 min and additional increase up to 80% acetonitrile in 0.1% formic acid over 5 min. This was followed by reduction in acetonitrile back to 2% and re-equilibration. The eluent was nebulised and ionised using the Thermo nano electrospray source with a distal coated fused silica emitter (New Objective, Woburn, MA, USA) with a capillary voltage of 1.8–2.2 kV. The Q Exactive instrument was operated in the data-dependent mode to automatically switch between full scan MS and MS/MS acquisition. Survey full scan MS spectra (*m*/*z* 375–1800) were acquired in the Orbitrap with 70 000 resolution (at *m*/*z* 200) after accumulation of ions to a 3 × 10^6^ target value with maximum injection time of 120 ms. Dynamic exclusion was set to 30 s. The 12 most intense multiply charged ions (*z*≥2) were sequentially isolated and fragmented in the octopole collision cell by higher-energy collisional dissociation (HCD) with a fixed injection time of 120 ms 17 500 resolution and AGC target of 1 × 10^5^ counts. A 2.0-Da isolation width was chosen. Under fill ratio was at 1% dynamic exclusion was set to 15 s. Mass spectrometric conditions were spray voltage, 2 kV; no sheath and auxiliary gas flow; heated capillary temperature, 275 °C; normalised HCD collision energy 27%. The mass spectrometry data were analysed online using X! tandem (release 2013.09.01; Craig and Beavis^61^) at the Global Proteome Machine site (www.thegpm.org). Raw data files were converted to peak lists in MGF format using MSconvert (ProteoWizard version 3.0.4922; Kessner *et al.*^62^) and were searched against Human proteins sequences (Ensemble GRC) appended with known contaminates (http://www.thegpm.org/cRAP/index.html) and mixed with reversed sequences. Search parameters include: trypsin cleavage specificity with two missed cleavage, cysteine carbamidomethyl as fixed modification, methionine oxidation and protein N-terminal acetylation as variable modifications. Peptide tolerance and MS/MS tolerance were set at 20 p.p.m. The remaining search parameters were default settings. The search results from the Global Proteome Machine site are provided in Worksheet 1, Supplementary Table 2.

### Statistical analysis

Statistical analysis was performed using GraphPad Prism v6 (GraphPad Software, Inc., La Jolla, CA, USA). Unless stated otherwise, experiments were performed on a minimum of three independent occasions. The statistical test applied to each data set is stipulated in the corresponding legend. A *P*-value of <0.05 was considered as statistically significant.

## Abbreviations

CSF: cerebrospinal fluid
DIC: differential interference contrast
DNA-PK: DNA-dependent protein kinase
EGFR: epidermal growth factor receptor
pEGFR: phosphorylated activate EGFR
GP: glycoprotein
PARP: poly ADP ribose polymerase
PRMs: platelet-released molecules
STS: staurosporine
veh: vehicle
SIN-1: linsidomine
OGD: oxygen-glucose deprivation
