# Supplementary figures and images for: Activated platelets rescue apoptotic cells via paracrine activation of EGFR and DNA-dependent protein kinase
Source: Cell Death Dis. 2014 Sep 11;5(9):e1410–. doi: 10.1038/cddis.2014.373 (PMC4540201; doi:10.1038/cddis.2014.373)

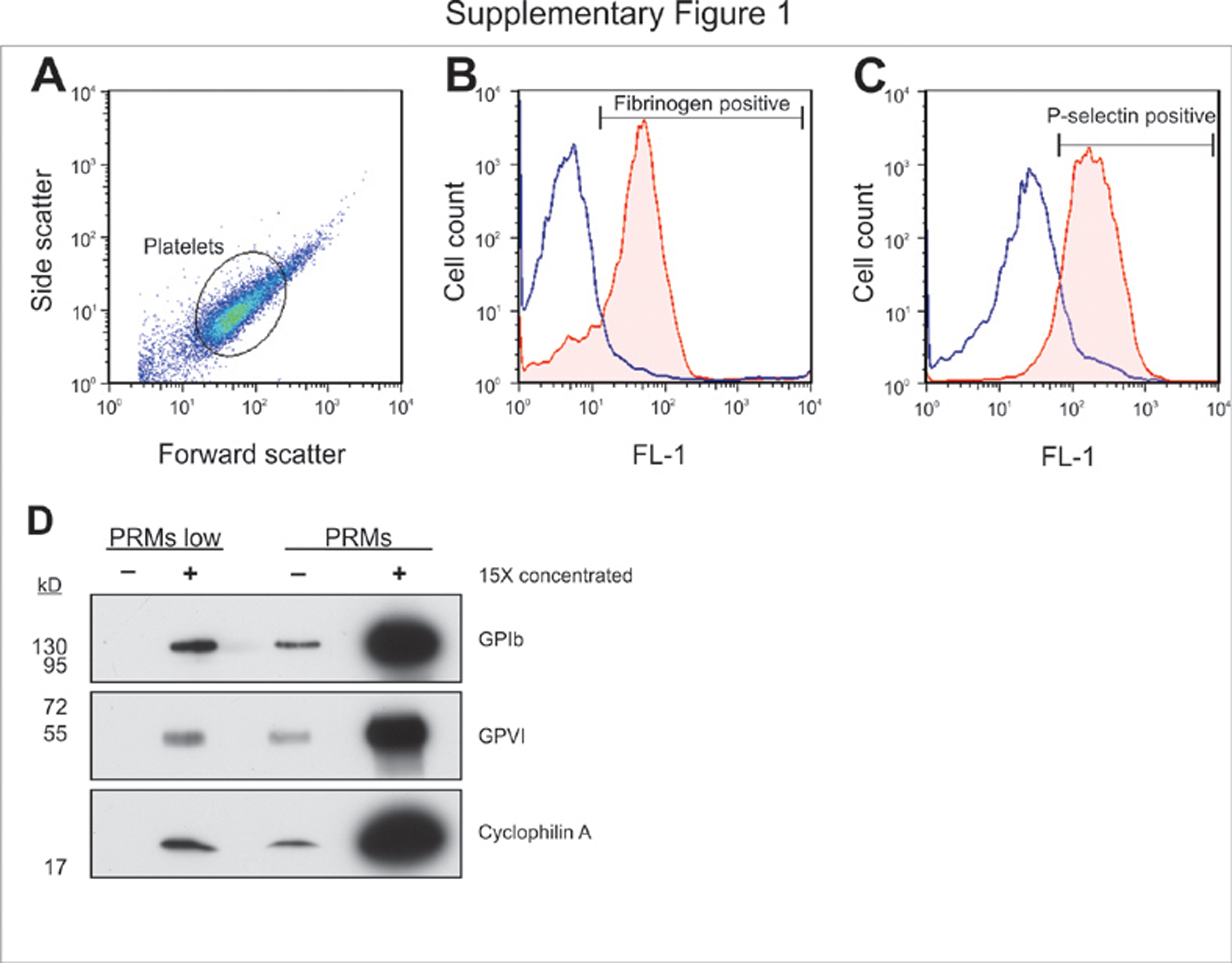

Supplement: Supplementary Figure 1 [file cddis2014373x1.tif]

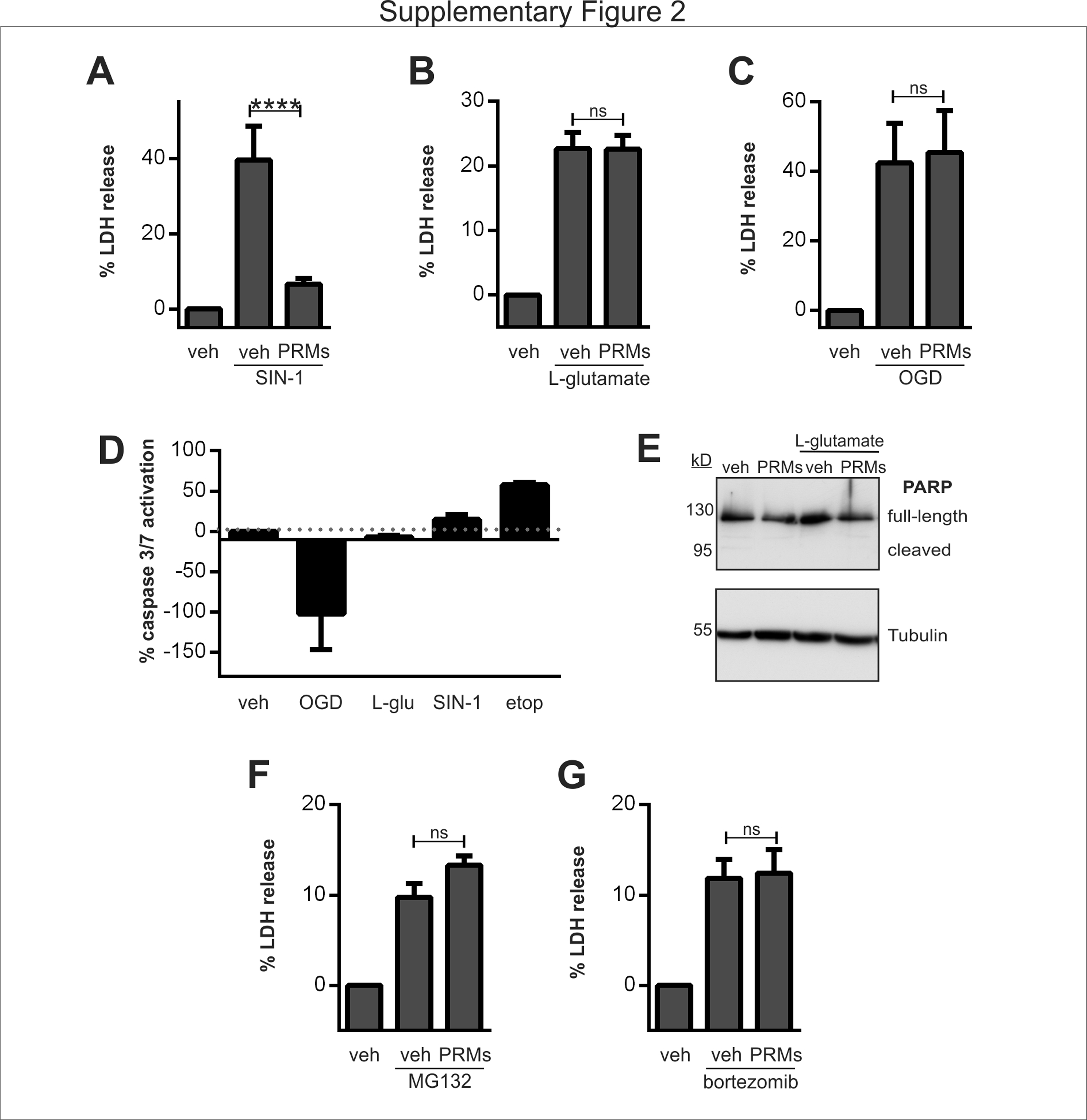

Supplement: Supplementary Figure 2 [file cddis2014373x2.tif]

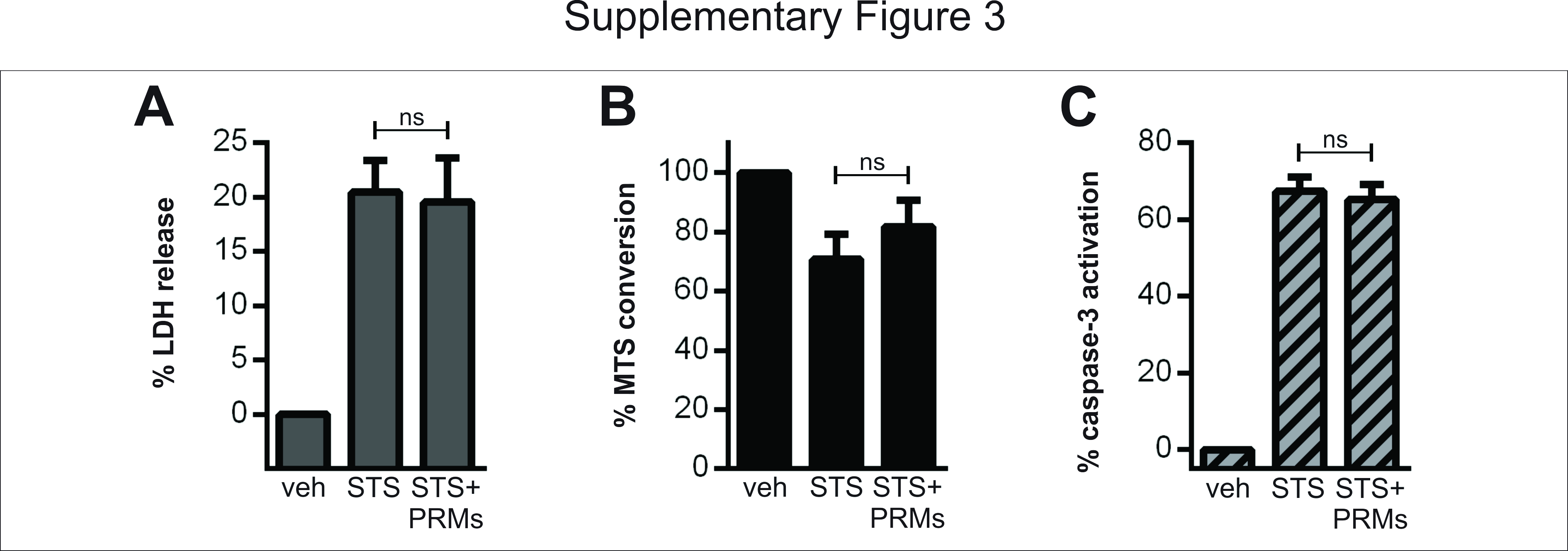

Supplement: Supplementary Figure 3 [file cddis2014373x3.tif]
